# Using Large Language Models to Assess the Consistency of Randomized Controlled Trials on AI Interventions With CONSORT-AI: Cross-Sectional Survey

**DOI:** 10.2196/72412

**Published:** 2025-09-26

**Authors:** Xufei Luo, Zeming Li, Zhenhua Yang, Bingyi Wang, Yanfang Ma, Fengxian Chen, Qi Wang, Long Ge, James Zou, Lu Zhang, Yaolong Chen, Zhaoxiang Bian

**Affiliations:** 1 Evidence-Based Medicine Center, School of Basic Medical Sciences, Lanzhou University, 199 Donggang West Road, Chengguan District, Lanzhou, 730000, China, 86 13893104140; 2 Research Unit of Evidence-Based Evaluation and Guidelines, Chinese Academy of Medical Sciences (2021RU017), School of Basic Medical Sciences, Lanzhou University, Lanzhou, China; 3 World Health Organization Collaboration Center for Guideline Implementation and Knowledge Translation, Lanzhou, China; 4 Institute of Health Data Science, Lanzhou University, Lanzhou, China; 5 Key Laboratory of Evidence-Based Medicine of Gansu Province, Lanzhou University, Lanzhou, China; 6 Department of Computer Science, Hong Kong Baptist University, Hong Kong, China (Hong Kong); 7 Vincent V.C. Woo Chinese Medicine Clinical Research Institute, School of Chinese Medicine, Hong Kong Baptist University, Hong Kong, China (Hong Kong); 8 Chinese EQUATOR Centre, Hong Kong, China (Hong Kong); 9 School of Information Science & Engineering, Lanzhou University, Lanzhou, China; 10 School of Nursing, Li Ka Shing Faculty of Medicine, University of Hong Kong, Hong Kong, China (Hong Kong); 11 Department of Health Research Methods, Evidence and Impact, Faculty of Health Sciences, McMaster University, Hamilton, ON, Canada; 12 Department of Health Policy and Management, School of Public Health, Lanzhou University, Lanzhou, China; 13 Department of Biomedical Data Science, Stanford University, Stanford, CA, United States; 14 Department of Electrical Engineering, Stanford University, Stanford, CA, United States; 15 Department of Computer Science, Stanford University, Stanford, CA, United States

**Keywords:** artificial intelligence, ChatGPT, CONSORT-AI, large language model, randomized controlled trials

## Abstract

**Background:**

Chatbots based on large language models (LLMs) have shown promise in evaluating the consistency of research. Previously, researchers used LLM to assess if randomized controlled trial (RCT) abstracts adhered to the CONSORT-Abstract guidelines. However, the consistency of artificial intelligence (AI) interventional RCTs aligning with the CONSORT-AI (Consolidated Standards of Reporting Trials-Artificial Intelligence) standards by LLMs remains unclear.

**Objective:**

The aim of this study is to identify the consistency of RCTs on AI interventions with CONSORT-AI using chatbots based on LLMs.

**Methods:**

This cross-sectional study employed 6 LLM models to assess the consistency of RCTs on AI interventions. The sample selection is based on articles published in *JAMA Network Open*, which included a total of 41 RCTs. All queries were submitted to LLMs through an application programming interface with a temperature setting of 0 to ensure deterministic responses. One researcher posed the questions to each model, while another independently verified the responses for validity before recording the results. The Overall Consistency Score (OCS), recall, inter-rater reliability, and consistency of contents were analyzed.

**Results:**

We found gpt-4‐0125-preview has the best average OCS on the basis of the results obtained by *JAMA Network Open* authors and by us (86.5%, 95% CI 82.5%‐90.5% and 81.6%, 95% CI 77.6%‐85.6%, respectively), followed by gpt-4‐1106-preview (80.3%, 95% CI 76.3%‐84.3% and 78.0%, 95% CI 74.0%‐82.0%, respectively). The model with the worst average OCS is gpt-3.5-turbo-0125 on the basis of the results obtained by *JAMA Network Open* authors and by us (61.9%, 95% CI 57.9%‐65.9% and 63.0%, 95% CI 59.0%‐67.0%, respectively). Among the 11 unique items of CONSORT-AI, Item 2 (“State the inclusion and exclusion criteria at the level of the input data”) received the poorest overall evaluation across the 6 models, with an average OCS of 48.8%. For other items, those with an average OCS greater than 80% across the 6 models included Items 1, 5, 8, and 9.

**Conclusions:**

GPT-4 variants demonstrate strong performance in assessing the consistency of RCTs with CONSORT-AI. Nonetheless, refining the prompts could enhance the precision and consistency of the outcomes. While AI tools like GPT-4 variants are valuable, they are not yet fully autonomous in addressing complex and nuanced tasks such as adherence to CONSORT-AI standards. Therefore, integrating AI with higher levels of human supervision and expertise will be crucial to ensuring more reliable and efficient evaluations, ultimately advancing the quality of medical research.

## Introduction

Transparent and standardized reporting of medical research is crucial for enhancing the quality and scientific integrity of studies [1]. Authors routinely adhere to reporting guidelines when drafting their articles to ensure comprehensive reporting. Widely adopted reporting standards encompass the Consolidated Standards of Reporting Trials (CONSORT) for randomized trials [2], Preferred Reporting Items for Systematic reviews and Meta-Analyses (PRISMA) for systematic reviews and meta-analyses [3], STrengthening the Reporting of OBservational studies in Epidemiology (STROBE) for observational studies [4], among others. These guidelines not only guide authors on how to comprehensively report the content of their studies but also serve as benchmarks for evaluating whether the reporting of published articles is complete and adheres to established guidelines. While these evaluations are relatively objective, the process can be quite time-intensive and lacks scalability.

The CONSORT-AI (Consolidated Standards of Reporting Trials-Artificial Intelligence) [5], introduced in 2020, aims to enhance transparency and thoroughness in the documentation of clinical trials involving AI interventions. Building upon the original CONSORT 2010 statement [2], this guideline extends the standards by mandating the routine inclusion of 11 additional items deemed critical for AI-based interventions. These additions emphasize the need for detailed reporting on AI intervention itself, including user guidelines, required competencies, the context of its application, and the management of its inputs and outputs. Moreover, CONSORT-AI underscores the importance of documenting the interaction between human users and the AI system, as well as a comprehensive analysis of instances where AI fails or errs. This level of detail is crucial for ensuring a clear understanding and evaluation of how the AI intervention is integrated and performs within a clinical setting. We focused on CONSORT-AI because it is the most widely recognized and accepted guideline for reporting AI-based randomized controlled trials (RCTs). Other guidelines were considered but excluded due to their lack of specificity or comprehensiveness for AI interventions. While our results may generalize to other frameworks, further research is needed to confirm this.

Large language models (LLMs) are advanced AI systems trained on massive corpus data, allowing them to understand and generate human-like text for a wide range of applications, from question-answering and content generation to language translation and evaluation [6]. LLMs have been increasingly applied in various fields beyond health care, such as education and social services, demonstrating their versatility and potential for automating complex tasks. For example, recent studies have explored the use of LLMs in educational settings to assist with personalized learning [7] and in social services to improve decision-making processes [8]. These applications highlight the broader relevance of AI-driven evaluations and the potential for LLMs to enhance efficiency and accuracy across multiple domains. Previous studies have suggested that LLMs or similar conversational AI models demonstrate promising performance in evaluating the risk of bias and quality of reporting [9-12]. However, no study has specifically examined the utility of employing LLMs to assess the adherence of RCTs involving AI interventions to the CONSORT-AI reporting guidelines. Given this gap, we conducted this study to investigate the performance of LLMs (GPT and Claude) [13,14] in evaluating the reporting quality of AI-related RCTs against the CONSORT-AI criteria. To our knowledge, this is the first study to systematically evaluate the performance of multiple LLMs in assessing the consistency of AI-based RCTs with the CONSORT-AI guidelines. By doing so, we provide a benchmark for the use of LLMs in this context and highlight their potential for automating the evaluation of reporting quality, which could significantly reduce the time and resource burden on human reviewers. Compared to existing tools like CONSORT-NLP, which is rule-based and effective for specific items but limited in adaptability to new guidelines or nuanced AI contexts [15], LLMs offer greater flexibility and scalability without requiring extensive retraining. Manual adherence checks are time-intensive, often taking hours per paper [10], highlighting the need for automated solutions like LLMs to alleviate this burden in real-world research evaluation.

## Methods

### Overview

In this study, we used a previously published systematic review as the benchmark for comparing the performance of LLMs [16]. The selected review evaluated 41 unique RCTs involving AI interventions against 11 key items from the CONSORT-AI reporting guidelines (Textbox 1). We selected this review because, firstly, it includes 41 RCTs of AI interventions, which is a relatively large sample size for this emerging field. Secondly, this review provides the evaluation results of each study from the CONSORT-AI guidelines, which serves as a convenient gold standard for evaluating the performance of LLMs. We did not prospectively register this study because it is a survey on LLMs, and whether it is registered or not does not affect the quality of the study or introduce bias. However, to enhance transparency, we have outlined a retrospective protocol including prompt refinement criteria, evaluation thresholds, and adjudication processes (see Multimedia Appendix 1 for details). We followed the Strengthening The Reporting Of Cohort Studies in Surgery (STROCSS) guidelines in writing and reporting this study [17]. For inter-rater reliability, one researcher posed the questions to each model, while another independently verified the responses for validity before recording the results; a third annotator reviewed 20% of the outputs for consistency, yielding a Cohen κ of 0.92.

Textbox 1. **CONSORT-AI (Consolidated Standards of Reporting Trials-Artificial Intelligence) unique checklist.**

1. Explain the intended use of the artificial intelligence (AI) intervention in the context of the clinical pathway, including its purpose and its intended users (eg, healthcare professionals, patients, public).

2. State the inclusion and exclusion criteria at the level of the input data.

3. Describe how the AI intervention was integrated into the trial setting, including any onsite or offsite requirements. Describe how the AI intervention was integrated into the trial setting, including any onsite or offsite requirements.

4. State which version of the AI algorithm was used. State which version of the AI algorithm was used.

5. Describe how the input data were acquired and selected for the AI intervention. Describe how the input data were acquired and selected for the AI intervention.

6. Describe how poor quality or unavailable input data were assessed and handled. Describe how poor quality or unavailable input data were assessed and handled.

7. Specify whether there was human-AI interaction in the handling of the input data, and what level of expertise was required of users.

8. Specify the output of the AI intervention. Specify the output of the AI intervention.

9. Explain how the AI intervention’s outputs contributed to decision-making or other elements of clinical practice.

10. Describe results of any analysis of performance errors and how errors were identified, where applicable. If such analysis was planned or done, justify why.

11. State whether and how the AI intervention and its code can be accessed, including any restrictions to access or re-use.

### Large Language Model Chatbots

We employed GPT-4 variants (gpt-4‐0125-preview and gpt-4‐1106-preview), ChatGPT 3.5 (gpt-3.5-turbo-0125 and gpt-3.5-turbo-1106), Claude-3-Opus-20240229, and Claude-3-sonnet-20240229 to evaluate the adherence of RCTs on AI intervention. We selected ChatGPT and Claude models due to their established performance in natural language understanding and generation tasks, particularly in complex domains such as medical research. These models have been widely used and validated in similar evaluative contexts, making them suitable for our study. While other models such as Gemini and LLaMA are also prominent, their inclusion would have increased the complexity of the study without necessarily providing additional insights, given the rapid evolution of LLMs and the focus of our research on well-established models [18,19].

### Prompt Engineering

Prompt engineering is crucial for generating accurate and concise outputs from LLMs by crafting well-designed instructions (prompts). We initiated the process by drafting an initial prompt based on ChatGPT’s prompt engineering guidelines [20], encompassing the LLM’s role, the task description, response rules, example responses, question descriptions, and the questions themselves. We then repeatedly tested and refined the prompt using GPT-4 variants on five RCTs until the results achieved an average accuracy rate of 85% or higher. The 5 articles used for prompt refinement were excluded from the final evaluation to prevent data leakage and ensure the validity of our results. After multiple rounds of testing, we finalized the prompt for ChatGPT. For example, the prompt template for ChatGPT was as follows: “You are an expert in evaluating the reporting quality of RCTs involving AI interventions. Based on the CONSORT-AI guidelines, assess whether the following RCT adheres to the specified items...”. Subsequently, we adapted this prompt for the Claude model with minor adjustments tailored to its architecture. Multimedia Appendix 2 presents the final prompts that we used to communicate with ChatGPT and Claude.

### The Process of Evaluation

**Figure 1.**
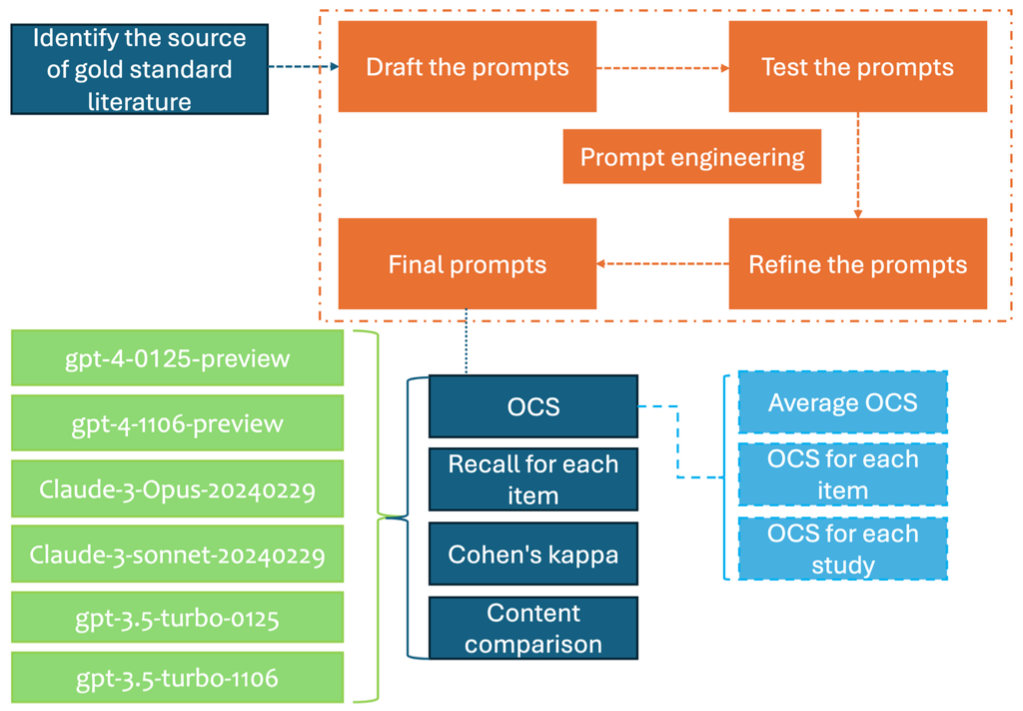
Flowchart of the study. OCS: Overall Consistency Score.

To facilitate the identification and evaluation of relevant information, we converted the 41 PDF documents into editable Word format using online conversion software Smallpdf . To save tokens, author names, institutions, and references were removed to reduce token usage and focus the LLMs on the core content of the RCTs. While this may have removed some contextual clues, we believe it did not significantly impact the assessment of CONSORT-AI items, as the key information is contained within the main text. No additional structured format was used, and truncation was not required as the input lengths were within the token limits of the models. All queries for each LLM were submitted through an application programming interface with a temperature setting of 0 to ensure deterministic responses. This setting minimizes variability in the output, which is crucial for maintaining consistency and accuracy in the evaluation of RCTs. Although the temperature was set to 0 to minimize variability, LLMs can still exhibit non-deterministic behavior due to factors such as hardware differences or model updates. One researcher posed the questions to each model, while another independently verified the responses for validity before recording the results. Verification involved ensuring that the LLM’s response was logically consistent and directly related to the input text. No responses were found to be invalid during this process. Each article was queried only once to maintain consistency. All results were documented in Excel for subsequent analysis. The entire querying process was conducted between April 8 and April 10, 2024. The process of the study is demonstrated in Figure 1.

### Data Analysis

Following the methodology we established, each constituent subgroup was subsequently scored and categorized into one of the two classifications (reported and not reported). We quantified consistency through an OCS, which reflects the proportion of criteria met, calculated as follows: OCS=(Number of items consistent with gold standards/11) × 100%. This yielded both an absolute score out of 11 (number of total items) and a percentage. The meaning of the OCS score is the percentage of items or studies where the results generated by the LLM match the gold standard we set. The OCS is a measure of accuracy, calculated as the proportion of items where the LLM’s assessment matches the gold standard. Content consistency, on the other hand, refers to the agreement between the specific excerpts extracted by the LLMs and those identified by human annotators. To further assess the quality of the data, we computed the Recall for each model, defined as the ratio of true positives—items correctly identified by the LLM from the full-text publication—to the sum of true positives and false positives. False positives represent instances where the LLMs generated data without any corresponding information in the full text (ie, “hallucinated data”) [21].

For inter-rater reliability, Cohen κ was employed to gauge the agreement between human responses and those generated by LLMs. The κ coefficient interpretation ranges as follows: 0.0-0.20 for slight agreement, 0.21-0.40 for fair agreement, 0.41-0.60 for moderate agreement, 0.61-0.80 for substantial agreement, and 0.81-1.0 for almost perfect agreement [22]. Meanwhile, to detect the accuracy of the LLM responses, we analyzed the consistency between the content extracted by GPT-4 variants and the content we extracted ourselves. The content we extracted was also used as one of the gold standards and compared with the standard from *JAMA Network Open*. Content consistency is defined as: (number of consistent items between the two/total number of items) × 100%. All statistical analyses were conducted using R (developed by Posit, version 2023.12.1 Build 402).

## Results

### Average Consistency Scores of Different Models

**Figure 2.**
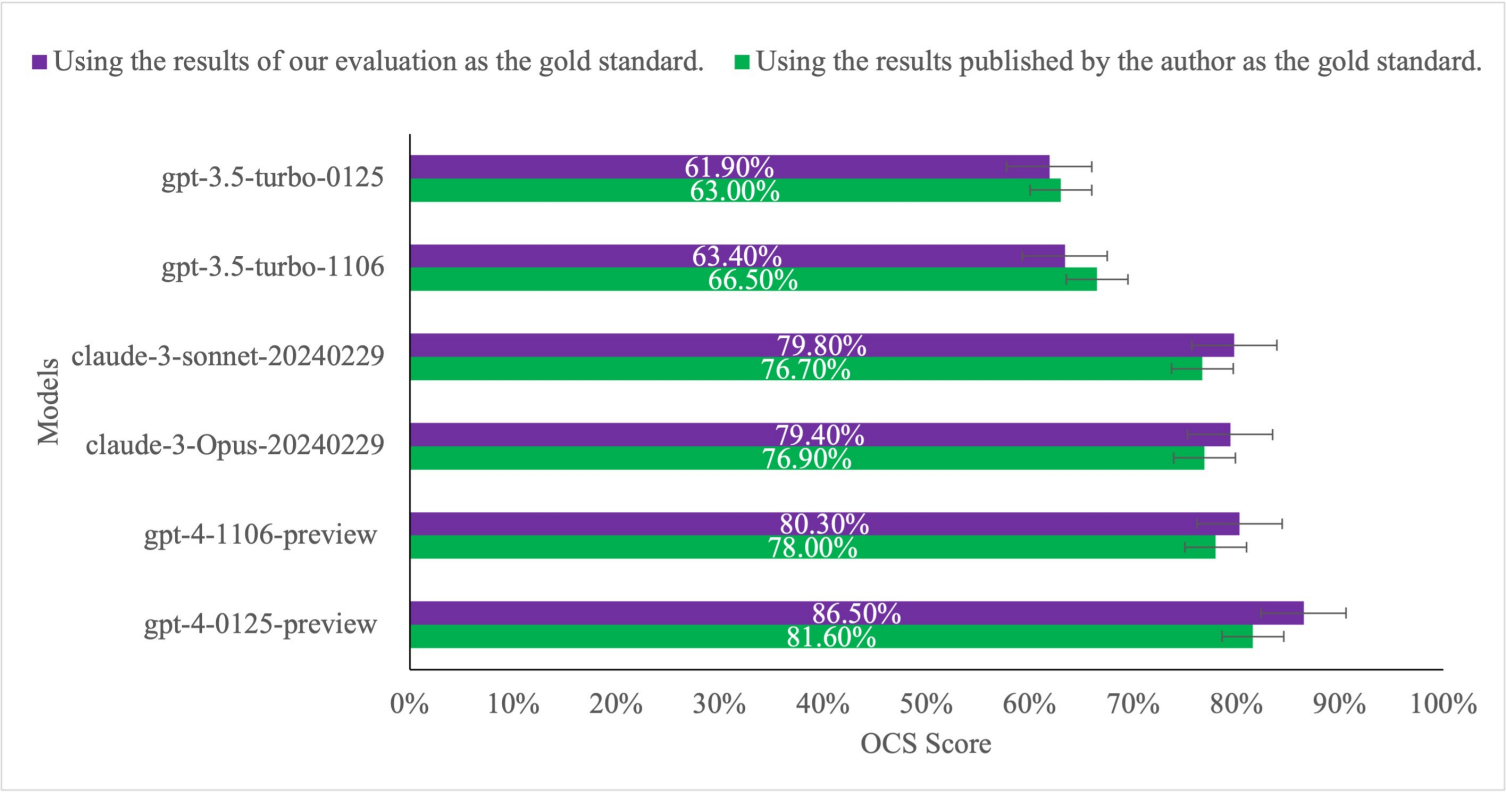
The average consistency score of different models. The green bars represent the evaluation results using *JAMA Network Open* authors as the gold standard, while the purple bars represent the results evaluated by us. The error bars represent the 95% CI.

The results from six different models revealed that GPT-4‐0125-preview had the highest average OCS, regardless of whether *JAMA Network Open* authors were used as the gold standard or our own evaluations were applied. Specifically, GPT-4‐0125-preview achieved an average OCS of 86.5% (95% CI 82.5%‐90.5%) according to *JAMA Network Open* authors, and 81.6% (95% CI 77.6%‐85.6%) based on our evaluations. Following this, GPT-4‐1106-preview had an average OCS of 80.3% (95% CI 76.3%‐84.3%) with *JAMA Network Open* authors as the gold standard, and 78.0% (95% CI 74.0%‐82.0%) based on our evaluations. The model with the worst average OCS is gpt-3.5-turbo-0125 (61.9%, 95% CI 57.9%‐65.9% and 63.0%, 95% CI 59.0%‐67.0%, respectively, as obtained by *JAMA Network Open* authors and us). ANOVA testing showed significant differences in OCS across models (*F*=21.48, *P<*.001), with post hoc Tukey HSD confirming GPT-4 variants outperformed Claude and GPT-3.5 (*P<*.05 for all pairwise comparisons). Specific average OCS values have been included in Figure 2. The detailed data are shown in Multimedia Appendix 3.

### Overall Consistency Score for Each Study

**Figure 3.**
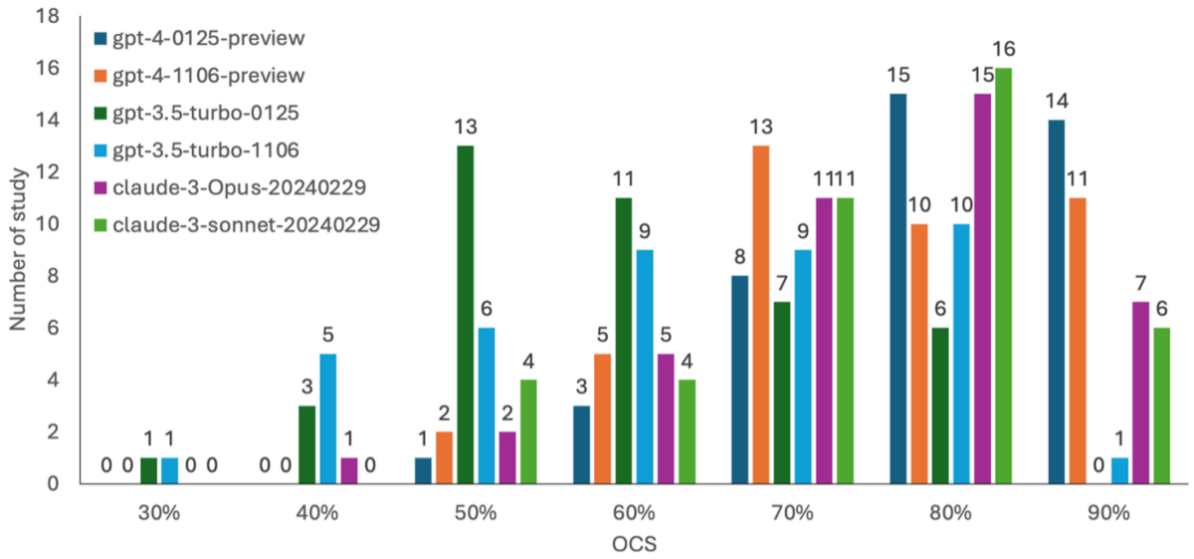
Histograms of Overall Consistency Score (OCS) scores from 41 randomized controlled trials using different models, with the *X*-axis representing OCS scores and the Y-axis representing frequencies or amount of study.

In the 41 RCTs included, the median OCS assessed by the gpt-4‐0125-preview was 81.8%, ranging from 54.5% to 100%. gpt-4‐1106-preview, claude-3-Opus-20240229, claude-3-sonnet-20240229, gpt-3.5-turbo-0125, and gpt-3.5-turbo-1106 were 72.7% (54.5%-100%), 81.8% (45.5%-100%), 81.8% (54.5%-100%), 63.6% (36.4%-81.8%), and 63.6% (36.4%-90.9%), respectively. The results are presented as a histogram in Figure 3. The specific results are shown in Multimedia Appendix 4.

### Overall Consistency Score for Each Item

**Figure 4.**
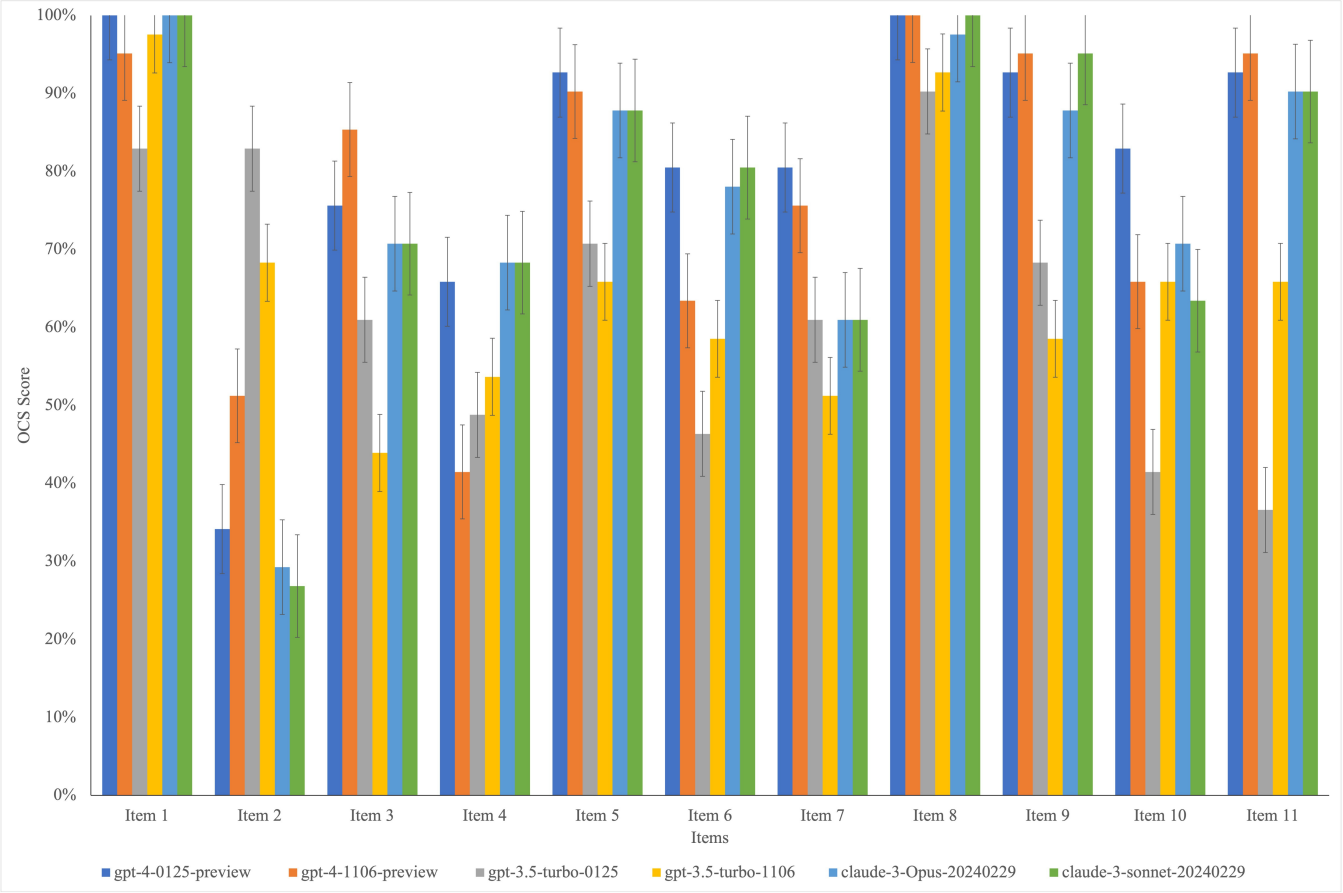
The overall consistency score for each item. The error bars represent the standard error.

Among the 11 unique items of CONSORT-AI, Item 2 (State the inclusion and exclusion criteria at the level of the input data) received the poorest overall evaluation across the 6 models, with an average OCS of 48.8%. For other items, those with an average OCS greater than 80% across the 6 models included Items 1, 5, 8, and 9, as detailed in Figure 4.

### Recall for Each Item

**Figure 5.**
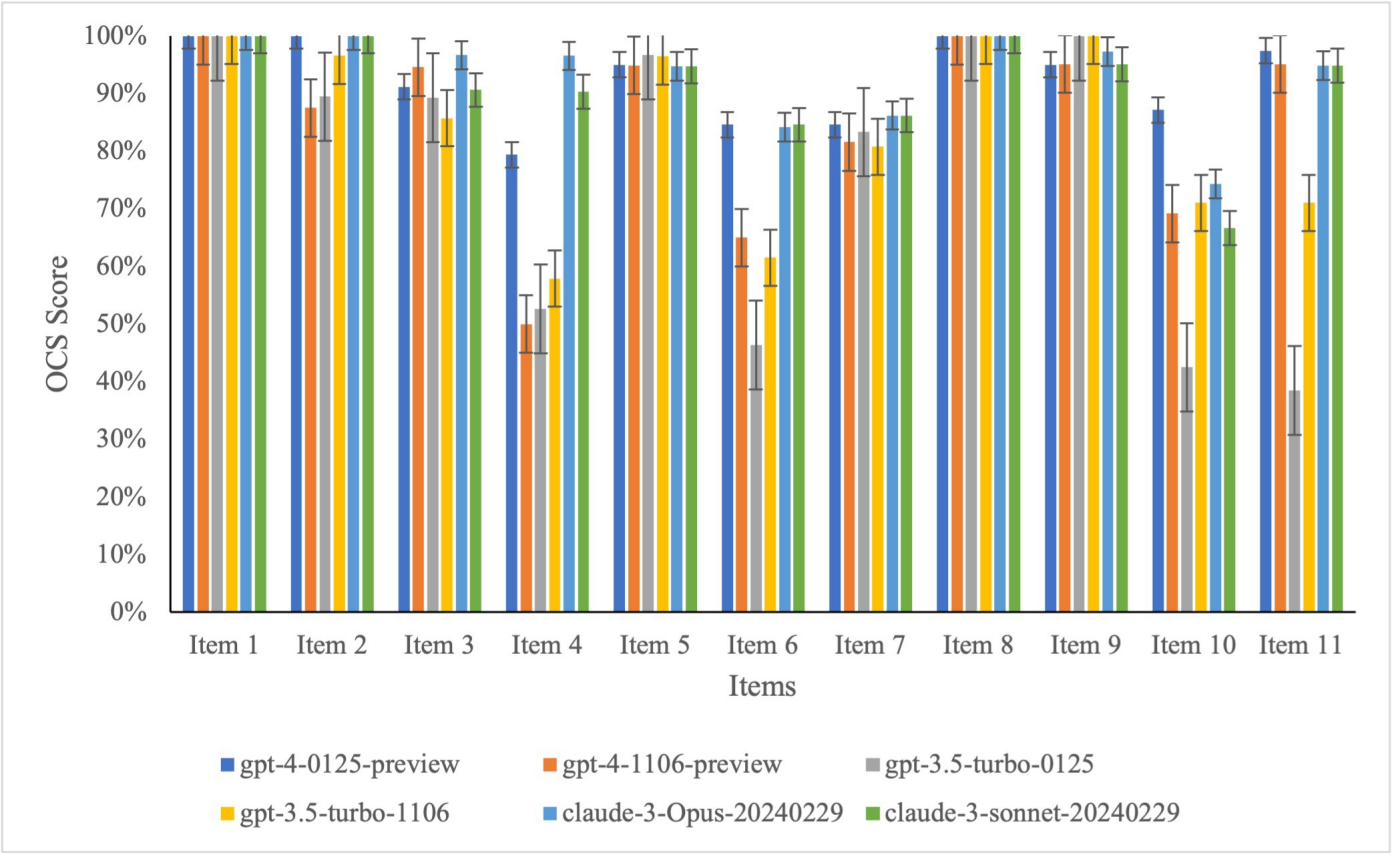
The recall for each item. The error bars represent the standard error.

The recall for each item varied. In CONSORT-AI, Items 1, 2, 3, 5, 7, and 8 show consistently high recall across the 6 different models, but the remaining 5 items showed significant variability in performance among different models, with overall lower recall, as shown in Figure 5.

### Consistency of Content Generated by GPT-4 Variants Compared to Humans

We reevaluated the included 41 AI intervention RCTs. We found inconsistencies between the content extracted by GPT-4‐0125-preview and our own extracted content. Consistency was defined as an exact match between the excerpt identified by the LLM and that identified by human annotators. No hallucinated quotes were observed in our analysis. In 41 studies, 29 (70.7%) studies showed inconsistencies; Item 2 had inconsistencies in 15 studies (36.6%), and Item 9 had inconsistencies in 11 studies (26.8%), as detailed in Multimedia Appendix 5. The agreement between our annotations and those from the systematic review was moderate, with a Cohen κ of 0.65, indicating that human evaluations also vary and providing context for the performance of the LLMs. Cohen κ values for comparisons between different models and the gold standard are presented in Multimedia Appendix 6.

## Discussion

### Principal Findings

We employed 3 different LLMs (ChatGPT4, ChatGPT 3.5, and Claude 3) from 6 different versions to evaluate the consistency of 41 AI intervention RCTs with CONSORT-AI. We found GPT-4 variants (gpt-4‐0125-preview) outperformed the other models, with significant variation in average OCS across different items, ranging from 26.8% to 100%, with an average score of 86.5%. The potential reason might be due to the varying levels of difficulty among different items, leading to different levels of understanding by the LLM tools. There is currently no recognized standard for what threshold indicates high consistency, but based on our experience, exceeding 85% suggests very good consistency. In addition, compared to the standard from *JAMA Network Open*, our own evaluation as the gold standard resulted in higher OCS scores in both GPT-4 variants and Claude. This suggests that there may be some variability in evaluation results between different individuals for the same item.

The highest consistency by GPT-4 variants (gpt-4‐0125-preview) could be attributed to its more refined understanding and processing capabilities, which are crucial in analyzing complex scientific texts. However, in some items (such as Items 2, 4, and 10) of the CONSORT-AI, all LLM chatbots perform poorly, possibly because these items are relatively complex. For example, in Item 2, GPT-4 misinterpreted data-level criteria as patient-level in one RCT we included, extracting “patients aged 18‐65” instead of “images with resolution >512 px,” likely due to prompt ambiguity around “input data.” Token limits were not an issue (average input <10k tokens), but model behavior suggests overfitting to common clinical phrases. The main reason might be that LLMs cannot effectively distinguish what the criteria are at the data level. Another example is Item 4, which requires reporting the version of the AI algorithm. However, many included studies report the time of algorithm development rather than the specific version, which we consider less precise as researchers may not always use the latest version. LLMs, however, cannot recognize this type of response as a report. Despite multiple adjustments and optimizations of the prompts, satisfactory results have not been achieved, indicating the need for future exploration of combining human annotation with LLMs to improve overall evaluation effectiveness. Another potential solution to this issue is to remove poorly performing items during checklist testing [23].

### Comparison to Prior Work

Compared to previous studies, which lacked specific focus on LLMs for evaluating AI RCT consistency with CONSORT-AI, our study provides a novel application of LLMs in this context. A similar study [15] conducted in 2020 developed the CONSORT-NLP tool, which used natural language processing methods to generate CONSORT checklists. The study indicated that the accuracy of 28 out of the 37 CONSORT items could reach over 90%. However, the CONSORT-NLP tool is rule-based and does not require retraining for different checklists, but it is limited to specific CONSORT items and may not be easily adaptable to other guidelines. Another study [10] used ChatGPT 3.5 to evaluate the adherence of RCT abstracts to the CONSORT-Abstract guidelines. The results demonstrated that ChatGPT can automate the appraisal of medical literature, facilitating the identification of accurately reported research. Additionally, our findings are consistent with those of Woelfle et al [24], Jiang et al [25], and Jiang et al [26], who also explored the use of AI in evaluating reporting quality. In contrast to the previous positive results, a study [9] using the RoB 2 tool to evaluate the risk of bias in RCTs indicated that ChatGPT and systematic reviewers only have “slight” to “fair” agreement in risk of bias judgments for randomized trials. Currently, ChatGPT is unable to reliably assess the risk of bias in randomized trials. More recent work by Changkai et al [27] on LLM assessment of RoB 2 further supports the potential of LLMs in evaluating research quality. Therefore, the evaluation results differ when using different models for different checklists. It is necessary to optimize and refine the prompts to achieve the best consistency with reporting guidelines for different types of study when using different checklists. The agreement between human annotators was moderate (Cohen κ=0.65), which is consistent with the findings from Woelfle et al [24] and provides context for interpreting LLM’s performance. Additionally, LLMs are known to generate hallucinated content, which can affect the reliability of their evaluations. To mitigate this, we set the temperature to 0 for deterministic responses and manually verified the responses for validity. Additionally, we calculated recall to assess the extent of hallucinated data, which were found to be minimal in our study.

### Future Directions

These findings underscore the need for continuous improvement in the reporting practices of AI intervention RCTs. LLMs like GPT-4 variants can play an important role in automating the evaluation of such checklists, potentially reducing the time and resource burden on human reviewers. The next step will be to optimize and refine the prompts for different reporting checklists recommended by the Enhancing the QUAlity and Transparency Of health Research (EQUATOR) Network based on various LLMs. This will be done to improve authors’ adherence to reporting guidelines and assist reviewers and journal editors in efficiently and quickly evaluating the completeness of manuscripts. To achieve this goal, future studies should focus on developing prompt engineering for different reporting guidelines and LLMs, performing testing and validation to achieve the best performance and outcomes. Given the large number of reporting checklists and the rapid evolution of LLMs, manual prompt refinement may be inefficient. Future research should explore automated or semiautomated methods for generating effective prompts.

Additionally, our study suggests that when using LLMs to evaluate the CONSORT-AI consistency of AI intervention RCTs, the results for Items 1, 5, 8, and 9 are reliable and recommended for use. However, the results for Item 2 are not reliable, and it is not recommended to use LLMs for this evaluation. The performance of other items may vary depending on different studies. For other types of RCTs, using LLMs to evaluate consistency with CONSORT or its extensions still requires further studies in the future.

### Strengths and Limitations

A key strength of this study is the comprehensive use of multiple closed-source LLM chatbots to evaluate a large number of RCTs, providing robust insights into the state of reporting consistency in the field of AI interventions. However, the study has some limitations. We found some inconsistencies between the results evaluated by the original authors and those evaluated by the LLMs. Therefore, we extracted specific contents from 41 RCTs, and the results indicated that some content was incorrectly evaluated by the authors, leading to these inconsistencies. We analyzed the consistency of the content, and it does not affect the overall conclusion. Moreover, the low adherence to certain items is mainly related to the difficulty in understanding the prompts and items, which requires continuous optimization and adjustment. Additionally, we recognize that these models have specific limitations compared to other approaches. For instance, although LLMs are valuable for initial content generation and consistency checks, they may lack the specialized decision-making capabilities or real-time adaptability that other models, such as those used in emergency triage settings[19], can offer. It will still be necessary to strengthen human supervision and enhance the capabilities of these models to better serve related medical tasks in the future. Additionally, considering the instability of the outputs of LLMs, we used only the results from the first output as the final results but conducted repeated runs on 10 RCTs, finding no variability in outputs due to the temperature setting of 0. Due to the rapid updates of LLMs, it is essential to update the models we use and the prompts in a timely manner to better suit the users. It is worth noting that this study used plain text input from Word documents. If users use PDF format documents for evaluation, the performance may be worse than the results presented in this study. Additionally, we acknowledge that our study relies on specific versions of LLMs (eg, ChatGPT and Claude), and this focus inherently limits the generalizability of the findings to other platforms and newer AI models. This limitation reflects the rapidly evolving nature of LLMs and the challenges of conducting such research within this dynamic landscape. Additionally, the study was not registered, which may limit transparency and reproducibility. Although registration is more common for clinical or observational studies, future research in this area could benefit from registering protocols to enhance methodological rigor. Moreover, although the studies were published before GPT-4’s knowledge cut-off, minimizing the risk of data leakage, future studies could benefit from using open-source models to further ensure no prior exposure to the data. Lastly, although 41 RCTs represent a relatively large sample for AI-based interventions, the small sample size may limit the generalizability of our findings. Future studies with larger datasets are needed to confirm our results.

In conclusion, while the use of LLMs demonstrates significant potential for helping in the consistency evaluation of AI intervention RCTs, there is still considerable room for improvement in both the tools and the reporting standards they are designed to assess. Current models have limitations, necessitating transparent versioning, bias detection mechanisms, and robust human oversight. Regulatory safeguards, such as audit trails and standards for AI in research assessment, are essential to prevent premature reliance. This study contributes to the ongoing dialog about the role of AI in enhancing the transparency and reliability of scientific reporting in health care.

## Supplementary material

10.2196/72412 Multimedia Appendix 1 Retrospective protocol for the study.

10.2196/72412 Multimedia Appendix 2 The prompts of ChatGPT and Claude.

10.2196/72412 Multimedia Appendix 3 Study adherence to CONSORT-AI guidelines.

10.2196/72412 Multimedia Appendix 4 The overall compliance scores of 41 included RCTs. The error bars represent the standard error.

10.2196/72412 Multimedia Appendix 5 Consistency of content generated by GPT-4 variants compared to human (a) by study, (b) by item.

10.2196/72412 Multimedia Appendix 6 Cohen κ values for comparisons between different models and the gold standard.

## Abbreviations

AI: artificial intelligence
CONSORT: Consolidated Standards of Reporting Trials
CONSORT-AI: Consolidated Standards of Reporting Trials-Artificial Intelligence
LLM: large language model
OCS: Overall Consistency Score
PRISMA: Systematic reviews and Meta-Analyses
RCT: randomized controlled trial
STROBE: Strengthening the Reporting of Observational studies in Epidemiology
