# Supplementary material for: Using Large Language Models to Assess the Consistency of Randomized Controlled Trials on AI Interventions With CONSORT-AI: Cross-Sectional Survey
Source: J Med Internet Res. 2025 Sep 26;27:e72412. doi: 10.2196/72412 (PMC12466798; doi:10.2196/72412)
Supplement: Multimedia Appendix 1 [file jmir-v27-e72412-s001.docx]

Appendix 1: Retrospective Protocol for the Study

Protocol Title

Using Large Language Models to Assess the Consistency of Randomized Controlled Trials on AI Interventions with CONSORT-AI: A Cross-Sectional Survey

Protocol Version——Version 1.0 (Retrospective, dated August 20, 2025)

Investigators

- Principal Investigators: James Zou, Ph.D.; Lu Zhang, Ph.D.; Yaolong Chen, M.D., Ph.D.

- Co-Investigators: Xufei Luo, M.P.H., Ph.D.; Zeming Li, Ph.D.; Zhenhua Yang, M.Sc(CompSc).; Bingyi Wang, M.P.H.; Yanfang Ma, M.Sc.; Fengxian Chen, Ph.D.; Qi Wang, Ph.D.; Long Ge, Ph.D.; Zhaoxiang Bian, M.D., Ph.D.

- On behalf of the ADVANCED working group

Funding and Support

Supported by the Vincent and Lily Woo Foundation and the Research Unit of Evidence-Based Evaluation and Guidelines, Chinese Academy of Medical Sciences (2021RU017), School of Basic Medical Sciences, Lanzhou University.

Background and Rationale

Transparent reporting in medical research, particularly for randomized controlled trials (RCTs) involving artificial intelligence (AI) interventions, is essential for ensuring reproducibility, reliability, and clinical applicability. The CONSORT-AI guidelines, an extension of the CONSORT 2010 statement, provide specific standards for reporting AI-based RCTs, including 11 unique items focused on AI-specific elements such as input data criteria, algorithm versioning, and error handling.

Large language models (LLMs) have emerged as powerful tools for automating complex tasks, including the evaluation of research reporting quality. Prior studies have demonstrated LLMs' utility in assessing adherence to guidelines like CONSORT-Abstract and risk of bias tools (e.g., RoB 2). However, no prior research has systematically evaluated LLMs for assessing CONSORT-AI adherence in AI-intervention RCTs.

This retrospective protocol describes the methods employed in our cross-sectional survey to evaluate six LLMs' performance in this context. The study addresses the gap by using a validated systematic review as a gold standard, aiming to benchmark LLM accuracy and identify areas for improvement. Given the exploratory nature of LLM surveys, prospective registration was not pursued initially; this retrospective protocol ensures transparency, mitigates potential biases (e.g., confirmation bias in prompt design), and facilitates reproducibility.

Objectives

To evaluate the consistency of assessments by six LLMs (GPT-4 variants, GPT-3.5 variants, and Claude-3 variants) with CONSORT-AI standards for 41 AI-intervention RCTs, using metrics including Overall Consistency Score (OCS), recall, inter-rater reliability (Cohen's kappa), and content consistency.

Secondary Objectives

1. To compare performance metrics across the six LLM models.

2. To identify CONSORT-AI items where LLMs exhibit poor performance and conduct error analysis.

3. To assess the impact of prompt engineering on LLM outputs and explore content discrepancies between LLM and human evaluations.

4. To discuss implications for automating reporting quality assessments in medical research.

Study Design

This is a cross-sectional survey design, conducted as a secondary analysis of published RCTs. The study adheres to the Strengthening the Reporting of Observational Studies in Epidemiology (STROBE) guidelines for cross-sectional studies and the STROCSS guidelines for reporting cohort, cross-sectional, and case-control studies in surgery (adapted for methodological surveys). No primary data collection from human participants was involved; ethical approval was not required.

The study was executed between March and April 2024, with data analysis completed by May 2024. This retrospective protocol aligns with the actual procedures performed.

Eligibility Criteria

Inclusion Criteria

- RCTs involving AI interventions, as identified in the systematic review by Plana et al. (2022) published in JAMA Network Open.

- Full-text articles available in PDF format.

- Evaluations based on the 11 unique items of the CONSORT-AI checklist (Table 1 in the manuscript).

Exclusion Criteria

- Non-RCT studies or those not focused on AI interventions.

- Five RCTs used exclusively for prompt refinement to avoid data leakage in the final evaluation.

Sample Size

A convenience sample of 41 RCTs from the referenced systematic review was used, providing a robust dataset for this emerging field without formal power calculations, as the study is exploratory.

Interventions (LLM Models and Procedures)

LLM Models

The following closed-source LLMs were selected based on their demonstrated performance in natural language tasks:

- GPT-4 variants: gpt-4-0125-preview and gpt-4-1106-preview (OpenAI).

- GPT-3.5 variants: gpt-3.5-turbo-0125 and gpt-3.5-turbo-1106 (OpenAI).

- Claude-3 variants: Claude-3-Opus-20240229 and Claude-3-Sonnet-20240229 (Anthropic).

Models were accessed via API interfaces with a temperature parameter set to 0 to ensure deterministic outputs and minimize variability.

Prompt Engineering

Prompts were developed iteratively to optimize LLM performance:

Initial Development: Prompts were crafted following OpenAI's prompt engineering best practices, including:

- Role assignment: "You are an expert in evaluating the reporting quality of RCTs involving AI interventions."

- Task description: Assess adherence to each of the 11 CONSORT-AI items (reported/not reported) with supporting excerpts.

- Response rules: Binary classification (reported/not reported), exact excerpts from text, no hallucinations.

- Examples: Provided for 2-3 items to illustrate expected outputs.

- **Refinement Process**: Tested on 5 excluded RCTs.

- Iteration Criteria: Adjustments made until average accuracy (compared to gold standard) reached ≥85%.

- Number of Iterations: 4 rounds, focusing on clarity for ambiguous items (e.g., distinguishing data-level vs. patient-level criteria in Item 2).

- Final Prompts: Model-specific adaptations (e.g., minor phrasing for Claude); full prompts documented in Appendix 1 of the manuscript.

Evaluation Procedures

1. Document Preparation: PDFs converted to editable Word format using Smallpdf (https://smallpdf.com). Removed non-essential elements (e.g., author names, references) to reduce token usage and focus on core content. No truncation was needed, as inputs were within model token limits.

2. Query Submission:

- Performed by one researcher (X.L.) via API.

- Each RCT queried once per model to maintain consistency.

- Repeated queries on a subset of 10 RCTs to assess output variability (expected to be zero due to temperature=0).

3. Output Verification and Adjudication:

- Independent review by a second researcher (Z.L.) for validity: Outputs must be logically consistent with RCT text, directly derived, and free of hallucinations.

- A third annotator (Z.Y.) reviewed 20% of outputs randomly selected for inter-rater agreement.

- Discrepancies resolved through consensus discussion among the three researchers.

- Gold Standards: (1) Evaluations from JAMA Network Open review; (2) Independent human re-evaluations by study team.

4. Timeline: Queries: April 8-10, 2024. Verification and analysis: April 11-20, 2024.

Outcome Measures

Primary Outcome

- Overall Consistency Score (OCS): Proportion of CONSORT-AI items where LLM assessment matches the gold standard, calculated as (Number of consistent items / 11) × 100%. Reported with 95% confidence intervals.

Secondary Outcomes

- Recall: True Positives / (True Positives + False Positives) per item, to quantify hallucination rates.

- Inter-Rater Reliability: Cohen's kappa between LLM and human evaluations, and among human annotators (interpretation: 0-0.20 slight, 0.21-0.40 fair, etc.).

- Content Consistency: Proportion of exact excerpt matches between LLM and human extractions.

- Item-Specific Performance: OCS and recall stratified by CONSORT-AI item.

- Error Analysis: Qualitative review of failure cases (e.g., misinterpretations in poor-performing items like Item 2).

Data Management and Analysis

Data Collection

- Outputs recorded in Excel spreadsheets, including binary classifications, excerpts, and verification notes.

- Human re-evaluations conducted blindly to JAMA standards.

Statistical Analysis

- Software: R version 2023.12.1 Build 402.

- Descriptive: Means, medians, ranges, histograms, boxplots (revised per JMIR figure policies).

- Inferential: ANOVA for inter-model OCS comparisons; post-hoc Tukey's HSD tests; p<0.05 significance level.

- Qualitative: Thematic analysis of inconsistencies (e.g., prompt ambiguity, model limitations).

- Subgroup Analyses: By model family (GPT vs. Claude) and gold standard.

Data Storage and Access

- Data stored securely on institutional servers at Lanzhou University.

- Analysis code available upon reasonable request to the corresponding authors.

Risk of Bias and Limitations Mitigation

- **Selection Bias**: Mitigated by using the full sample from a published review.

- **Confirmation Bias**: Predefined refinement thresholds (≥85% accuracy) and independent adjudication.

- **Variability in LLMs**: Temperature=0; subset repeated queries to confirm determinism.

- **Hallucinations**: Measured via recall; manual verification ensured no invalid outputs.

- **Generalizability**: Limited to selected models and RCTs; discussed in manuscript limitations.

Ethical Considerations

- No human subjects or sensitive data involved.

- All RCTs were publicly available publications.

- Conflicts of Interest: None declared.

- No generative AI used in manuscript writing.

Dissemination

Results to be published in peer-reviewed journals (e.g., JMIR). Protocol shared as Appendix 5 in the manuscript.
