# Supplementary material for: Using Large Language Models to Assess the Consistency of Randomized Controlled Trials on AI Interventions With CONSORT-AI: Cross-Sectional Survey
Source: J Med Internet Res. 2025 Sep 26;27:e72412. doi: 10.2196/72412 (PMC12466798; doi:10.2196/72412)
Supplement: Multimedia Appendix 2 [file jmir-v27-e72412-s002.docx]

**Appendix 2 The prompts of ChatGPT and Claude**

**Your role**:

Suppose you are a clinical trial author and evaluator specializing in AI interventions.

**Your task**:

Your task is to conduct a thorough critical appraisal of the clinical trial on AI interventions presented in the submitted article. Your insights will contribute to an in-depth understanding of the trial's quality and the reliability of its results. Please utilizing your expertise, respond to the questions listed in the following **Questions part** based on the following **Response rules** and **Question descriptions**. **Question descriptions** add additional detailed descriptions to the questions listed in **Questions part**.

**Response rules**:

1.

If the answer to the question is reported or presented in the paper, reply with 1; otherwise, reply with 0.

2.

For questions to which you reply with ‘0’, please give your detailed explanation. For questions to which you reply with ‘1’, please list the original and unmodified content from the uploaded article in an ascending order, formatted as 1, 2, 3, etc.

3.

Each response should be informed and supported by the question description, ensuring a clear connection between the question asked and the description given.

**Response Example**:

Question 1):

Answer: 0/1

Original and unmodified content from the uploaded article:

1. xxxxxx.

2. xxxxxx.

Etc.

**Question descriptions**:

The description for Question 1):

In order to clarify how the AI intervention is intended to fit into a clinical pathway, a detailed description of its role should be included in the background of the trial report. AI interventions may be designed to interact with different users, including healthcare professionals, patients, and the public, and their roles can be wide-ranging (for example, the same AI intervention could theoretically be replacing, augmenting, or adjudicating components of clinical decision-making). Clarifying the intended use of the AI intervention and its intended user helps readers understand the purpose for which the AI intervention was evaluated in the trial.

The description for Question 2):

It is important to note that the exclusion criteria at the input data level are based on the data, NOT on patient age, sex, disease and others, ‘Input data’ refers to the data required by the AI intervention to serve its purpose (for example, for a breast cancer diagnostic system, the input data could be the unprocessed or vendor-specific post-processing mammography scan upon which a diagnosis is being made; for an early-warning system, the input data could be physiological measurements or laboratory results from the electronic health record). The trial report should pre-specify if there were minimum requirements for the input data (such as image resolution, quality metrics, or data format) that determined pre-randomization eligibility. It should specify when, how and by whom this was assessed. For example, if a participant met the eligibility criteria for lying flat for a CT scan, but the scan quality was compromised (for any given reason) to such a level that it was deemed unfit for use by the AI system, this should be reported as an exclusion criterion at the input-data level. Note that where input data are acquired after randomization, any exclusion is considered to be from the analysis, not from enrollment.

The description for Question 3):

There are limitations to the generalizability of AI algorithms, one of which is when they are used outside of their development environment. AI systems depend on their operational environment, and the report should provide details of the hardware and software requirements to allow technical integration of the AI intervention at each study site. For example, it should be stated if the AI intervention required vendor-specific devices, if there was specialized computing hardware at each site, or if the site had to support cloud integration, particularly if this was vendor specific. If any changes to the algorithm were required at each study site as part of the implementation procedure (such as fine-tuning the algorithm on local data), then this process should also be clearly described.

The description for Question 4):

Similar to other forms of software as a medical device, AI systems are likely to undergo multiple iterations and updates during their lifespan. It is therefore important to specify which version of the AI system was used in the clinical trial, whether this is the same as the version evaluated in previous studies that have been used to justify the study rationale, and whether the version changed during the conduct of the trial. If applicable, the report should describe what has changed between the relevant versions and the rationales for the changes. Where available, the report should include a regulatory marking reference, such as an unique device identifier, that requires a new identifier for updated versions of the device.

The description for Question 5):

The measured performance of any AI system may be critically dependent on the nature and quality of the input data. A description of the input-data handling, including acquisition, selection and pre-processing before analysis by the AI system, should be provided.

The description for Question 6):

‘input data’ refers to the data required by the AI intervention to serve its purpose. The performance of AI systems may be compromised as a result of poor quality or missing input data (for example, excessive movement artifact on an electrocardiogram). The trial report should report the amount of missing data, as well as how this was identified and handled. The report should also specify if there was a minimum standard required for the input data and, where this standard was not achieved, how this was handled (including the impact on, or any changes to, the participant care pathway).

The description for Question 7):

A description of the human–AI interface and the requirements for successful interaction when input data are handled should be provided — for example, clinician-led selection of regions of interest from a histology slide that is then interpreted by an AI diagnostic system, or an endoscopist’s selection of a colonoscopy video clips as input data for an algorithm designed to detect polyps. A description of any user training provided and instructions for how users should handle the input data provides transparency and replicability of trial procedures.

The description for Question 8):

The output of the AI intervention should be clearly specified in the trial report. For example, an AI system may output a diagnostic classification or probability, a recommended action, an alarm alerting to an event, an instigated action in a closed-loop system (such as titration of drug infusions) or another output.

The description for Question 9):

Since health outcomes may also critically depend on how humans interact with the AI intervention, the report should explain how the outputs of the AI system were used to contribute to decision-making or other elements of clinical practice. This should include adequate description of downstream interventions that can affect outcomes. Any effects of human–AI interaction on the outputs should be described in detail, including the level of expertise required to understand the outputs and any training and/or instructions provided for this purpose. For example, a skin cancer detection system that produced a percentage likelihood as its output should be accompanied by an explanation of how this output was interpreted and acted upon by the user, specifying both the intended pathways (for example, skin lesion excision if the diagnosis is positive) and the thresholds for entry to these pathways (for example, skin lesion excision if the diagnosis is positive and the probability is >80%). The information produced by comparator interventions should be similarly described, alongside an explanation of how such information was used to arrive at clinical decisions on patient management, where relevant. Any discrepancy in how decision-making occurred versus how it was intended to occur (that is, as specified in the trial protocol) should be reported.

The description for Question 10):

Reporting performance errors and failure case analysis is especially important for AI interventions. AI systems can make errors that may be hard to foresee but that, if allowed to be deployed at scale, could have catastrophic consequences. Therefore, reporting cases of error and defining risk-mitigation strategies are important for informing when, and for which populations, the intervention can be safely implemented. The results of any performance-error analysis should be reported and the implications of the results should be discussed.

The description for Question 11):

The trial report should make it clear whether and how the AI intervention and/or its code can be accessed or re-used. This should include details about the license and any restrictions to access.

**Questions part**:

Question 1):

Explain the intended use for the AI intervention in the context of the clinical pathway, including its purpose and its intended users (for example, healthcare professionals, patients, and the public).

Question 2):

State the inclusion and exclusion criteria of the input data.

Question 3):

Describe how the AI intervention was integrated into the trial setting, including any onsite or offsite requirements.

Question 4):

State which version of the AI algorithm was used.

Question 5):

Describe how the input data were acquired and selected for the AI intervention.

Question 6):

Describe how poor-quality or unavailable input data were assessed and handled.

Question 7):

Specify whether there was human–AI interaction in the handling of the input data, and what level of expertise was required of users.

Question 8):

Specify the output of the AI intervention.

Question 9):

Explain how the AI intervention’s outputs contributed to decision-making or other elements of clinical practice.

Question 10):

Describe results of any analysis of performance errors and how errors were identified, where applicable. If no such analysis was planned or done, justify why not.

Question 11):

State whether and how the AI intervention and/or its code can be accessed, including any restrictions to access or re-use.

**Answers**:

Each question listed in **Questions part** has corresponding additional detailed description in **Question descriptions**.

Please answer the questions follow the **Response rules** and **Response Example**. Response must start with ```start and end with ```end, follows the "Response Example".
