# Supplementary material for: Using Large Language Models to Assess the Consistency of Randomized Controlled Trials on AI Interventions With CONSORT-AI: Cross-Sectional Survey
Source: J Med Internet Res. 2025 Sep 26;27:e72412. doi: 10.2196/72412 (PMC12466798; doi:10.2196/72412)
Supplement: Multimedia Appendix 5 [file jmir-v27-e72412-s005.docx]

**Appendix 5** Consistency of content generated by GPT-4 variants compared to human (a) by study, (b) by item.

(a)

(b)
