# Supplementary material for: Using Large Language Models to Assess the Consistency of Randomized Controlled Trials on AI Interventions With CONSORT-AI: Cross-Sectional Survey
Source: J Med Internet Res. 2025 Sep 26;27:e72412. doi: 10.2196/72412 (PMC12466798; doi:10.2196/72412)
Supplement: Multimedia Appendix 6 [file jmir-v27-e72412-s006.docx]

**Appendix 6 Cohen's kappa values for comparisons between different models and the gold standard.**

| **Items** | **Cohen's kappa, 95%CI, *P* value** | | | | | |
| --- | --- | --- | --- | --- | --- | --- |
|  | **Vs. gpt-4-0125-preview** | **Vs. gpt-4-1106-preview** | **claude-3-Opus-20240229** | **claude-3-sonnet-20240229** | **gpt-3.5-turbo-0125** | **gpt-3.5-turbo-1106** |
| 1 | 1.00, (95%CI: 1.0 - 1.0, *P*<0.01) | 1.00, (95%CI: 1.0 - 1.0, *P*<0.01) | 1.00, (95%CI: 1.0 - 1.0, *P*<0.01) | 1.00, (95%CI: 1.0 - 1.0, *P*<0.01) | 0.089, (95%CI: -0.274, 0.452, *P*=0.569) | 1.00, (95%CI: 1.0 - 1.0, *P*<0.01) |
| 2 | 0.075, (95%CI: -0.002, 0.153, *P*=0.206) | -0.033, (95%CI: -0.242, 0.177, *P*=0.762) | 0.007, (95%CI: -0.107, 0.120, *P*=0.910) | 0.038, (95%CI: -0.008, 0.083, *P*=0.374) | 0.089, (95%CI: -0.274, 0.452, *P*=0.569) | 0.240, (95%CI: -0.013, 0.492, *P*<0.05) |
| 3 | 0.235, (95%CI: -0.103，0.573, *P=*0.114) | 0.485, (95%CI: 0.134, 0.836, *P*=0.002) | 0.245, (95%CI: -0.043, 0.534, *P*=0.069) | 0.057, (95%CI: -0.244, 0.358, *P*=0.697) | 0.076, (95%CI: -0.182, 0.334, *P*=0.551) | -0.033, (95%CI: -0.233, 0.167, *P*=0.745) |
| 4 | 0.137, (95%CI: -0.177，0.452, *P*=0.378) | -0.121, (95%CI: -0.394, 0.152, *P*=0.382) | 0.053, (95%CI: -0.153,0.260, *P*=0.569) | 0.229, (95%CI: -0.078, 0.536, *P*=0.112) | 0.097, (95%CI: -0.128, 0.321, *P*=0.418) | 0.156, (95%CI: -0.082, 0.394, *P*=0.221) |
| 5 | -0.034, (95%CI: -0.081, 0.013, *P*=0.819) | -0.051, (95%CI: -0.101, -0.001, *P*=0.743) | -0.062, (95%CI: -0.121, -0.003, *P*=0.684) | -0.062, (95%CI: -0.121, -0.003, *P*=0.684) | 0.065, (95%CI: -0.158, 0.288, *P*=0.509) | 0.043, (95%CI: -0.148, 0.235, *P*=0.628) |
| 6 | 0.109, (95%CI: -0.235, 0.453, *P*=0.437) | 0.100, (95%CI: -0.109, 0.308, *P*=0.308) | 0.109, (95%CI: -0.235, 0.453, *P*=0.437) | 0.109, (95%CI: -0.235, 0.453, *P*=0.437) | 0.096, (95%CI: -0.014, 0.206, *P*=0.150) | -0.020, (95%CI: -0.207, 0.166, *P*=0.834) |
| 7 | 0.388, (95%CI: 0.052, 0.724, *P*<0.01) | 0.235, (95%CI: -0.103, 0.573, *P*=0.114) | 0.138, (95%CI: -0.134, 0.411, *P*=0.319) | 0.168, (95%CI: -0.113, 0.448, *P*=0.238) | 0.121, (95%CI: -0.174, 0.416, *P*=0.413) | 0.012, (95%CI: -0.254, 0.278, *P*=0.929) |
| 8 | 1.00, (95%CI: 1.0 - 1.0, *P*<0.01) | 1.00, (95%CI: 1.0 - 1.0, *P*<0.01) | 1.00, (95%CI: 1.0 - 1.0, *P*<0.01) | 1.00, (95%CI: 1.0 - 1.0, *P*<0.01) | -0.051, (95%CI: -0.101, -0.001, *P*=0.743) | -0.051, (95%CI: -0.101, -0.001, *P*=0.743) |
| 9 | -0.034, (95%CI: -0.081,0.013, *P*=0.819) | -0.034, (95%CI: -0.081,0.013, *P*=0.819) | 0.461, (95%CI:0.038, 0.883, *P*<0.01) | 0.773, (95%CI: 0.475,1.00, *P*<0.01) | 0.163, (95%CI: -0.043, 0.369, *P*=0.056) | 0.112, (95%CI: -0.036, 0.261, *P*=0.119) |
| 10 | 0.288, (95%CI: -0.080,0.655, *P*<0.05) | -0.003, (95%CI: -0.209, 0.203, *P=0.975*) | 0.031, (95%CI: -0.217, 0.280, *P*=0.792) | -0.003, (95%CI: -0.209, 0.203, *P*=0.975) | 0.014, (95%CI: -0.114, 0.142, *P*=0.834) | -0.130, (95%CI: -0.254, -0.006, *P*=0.276) |
| 11 | 0.532, (95%CI: 0.068, 0.997, *P*<0.01) | 0.773, (95%CI: 0.475,1.00, *P*<0.01) | 0.446, (95%CI: -0.009, 0.901, *P*=0.004) | 0.446, (95%CI: -0.009, 0.901, *P*=0.004) | -0.043, (95%CI: -0.197, 0.111, *P*=0.558) | -0.025, (95%CI: -0.263, 0.213, *P*=0.843) |
